# Roles of Cysteine Proteases in Biology and Pathogenesis of Parasites

**DOI:** 10.3390/microorganisms11061397

**Published:** 2023-05-26

**Authors:** Nawu Yang, Maurice A. Matthew, Chaoqun Yao

**Affiliations:** Department of Biomedical Sciences and One Health Center for Zoonoses and Tropical Veterinary Medicine, Ross University School of Veterinary Medicine, Basseterre P.O. Box 334, Saint Kitts and Nevis; nawuyang@students.rossu.edu (N.Y.); mmatthew@rossvet.edu.kn (M.A.M.)

**Keywords:** cysteine proteases, thiol proteases, parasites, virulence, diagnosis, chemotherapy

## Abstract

Cysteine proteases, also known as thiol proteases, are a class of nucleophilic proteolytic enzymes containing cysteine residues in the enzymatic domain. These proteases generally play a pivotal role in many biological reactions, such as catabolic functions and protein processing, in all living organisms. They specifically take part in many important biological processes, especially in the absorption of nutrients, invasion, virulence, and immune evasion of parasitic organisms from unicellular protozoa to multicellular helminths. They can also be used as parasite diagnostic antigens and targets for gene modification and chemotherapy, as well as vaccine candidates, due to their species and even life-cycle stage specificity. This article highlights current knowledge on parasitic cysteine protease types, biological functions, and their applications in immunodiagnosis and chemotherapy.

## 1. Introduction

**Figure 1:**
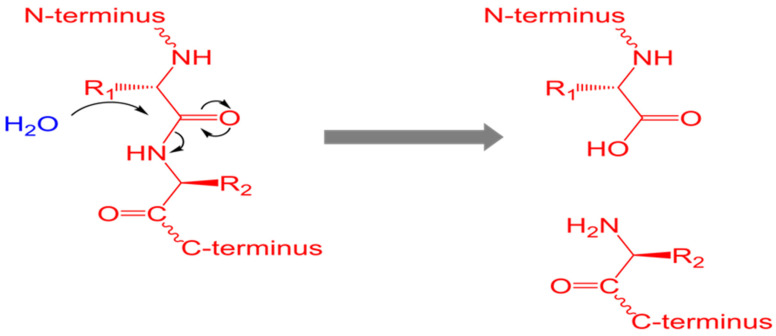
The hydrolysis of a protein via the nucleophilic attack of water. Proteolysis breaks down proteins into smaller polypeptides or individual amino acids. Proteolysis is typically catalyzed by cellular enzymes called proteases but may also occur by intra-molecular digestion (Thomas Shafee, CC BY 4.0 <https://creativecommons.org/licenses/by/4.0, accessed on 3 April 2023>, via Wikimedia Commons).

Proteases, which are localized in different parts of a cell, including the plasma membrane, cytoplasm, or even excretory-secretory proteins (ESP), act as simple yet powerful destructive enzymes necessary for protein catabolism and amino acid (AA) production in all living organisms (Figure 1). However, in addition to these degradation functions, proteases act as sharp scissors and catalyze highly specific reactions of proteolytic processing, resulting in new peptides/protein products [1]. Moreover, proteases play a critical role in many biological, physiological and pathophysiological processes in the majority of unicellular and multicellular parasites [2,3,4].

Based on their catalytic mechanism, proteases are classified into six distinct classes, i.e., metalloproteases, aspartic, glutamic, cysteine, serine, and threonine proteases [5]. Among them, cysteine protease (CP) is a secreted protein widely present in various parasites, involved in a variety of biological and pathogenetic processes such as adherence to host cells, tissue invasion, cytotoxicity, nutrient uptake, and immune evasion of parasites. Further, CP often has strong antigenicity [6]. The aims of this article were to provide a fundamental platform for and highlight CPs’ biological functions and their applications in immunodiagnosis and chemotherapy of endoparasites from unicellular protozoa to multicellular helminths.

## 2. Classification of Cysteine Proteases

**Figure 2:**
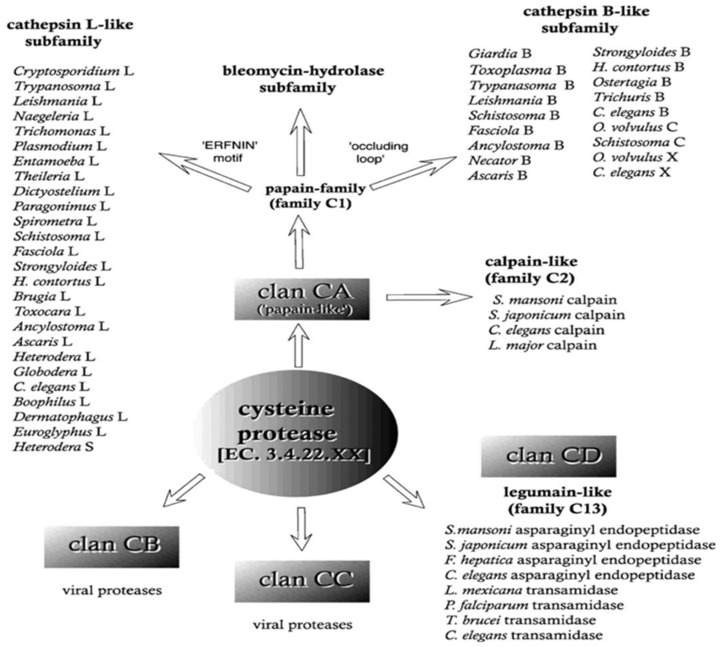
Schematic diagram of the cysteine protease subfamilies, which highlights how cysteine proteases of parasitic organisms are related to one another. Subfamilies are determined by homology in amino acid sequence at the enzymatic active site [23]. Reprinted from reference [23] with permission of Elsevier).

CPs are relatively conservative during evolution, and these eosinophilic proteolytic enzymes have the highest hydrolytic activity at the optimal pH 4~6.5. They are classified into various families according to the primary AA sequence and secondary/tertiary structure. Families thought to have a common evolutionary origin are grouped as clans [7]. There are 10 clans of CPs, namely CA, CB, CC CD, CE, CF, CL, CM, CN, and CO. Among all ten clans, there are a total of 113 families, from C1 to C124 [8]. Each family is further divided into different subfamilies, which are determined by homology in AA sequence at the enzymatic active site (Figure 2). CPs in the same clans share similar catalytic mechanisms by using indispensable cysteine residues, although they may have no sequence identity and differ in secondary/tertiary structure. In this context, the catalytic cysteine of the CPs is biologically significant because it performs hydrolysis in all cases. The functions of CP’s hydrolysis always involve the catalytic triad of Cys/His/Asn (or Asp). In addition, the 4th residue is usually Gln. The latter is important for stabilizing the acyl intermediates formed during the catalytic process and takes precedence over Cys activity sites [9,10]. 

Most CPs of parasitic protozoa that have already been characterized so far belong to the clan CA [11], a clan that is represented by papain-like proteases. It also includes cathepsin B, H, K, S and cathepsin L-like proteases and the C2 family (calpain-like), with a leader peptide [12] (Figure 2). Members of the clan CA are generally sensitive to the small molecule inhibitor trans-epoxysuccinyl-l-leuciloamido-(4-guanidino) butane (E-64), which is ineffective against CPs in other clans. 

Many enzymes in the clan CD have been isolated from parasitic protozoa and have important functions that are completely different from their known mammalian counterparts [13]. The main members of this clan are asparaginyl endopeptidase with a glycosylphosphatidylinositol (GPI) anchor that attaches a protein to the plasma membrane (Figure 2, C13 family). GPI anchors many surface proteins to the plasma membrane of eukaryotic cells. Interestingly, the annotated genome of *Trichomonas vaginalis* does not predict any genes for GPI anchors [14], which is surprising as GPIs are important and abundant glycolipids used by many parasitic protists to anchor molecules on the cell surface, including virulent factors [15]. The C11 family is known as clostripain [16]; C14, caspase; C25, gingipain; and C50, separases [17,18,19]. Asparaginyl endopeptidases specifically hydrolyze the carboxy-terminal peptide bond of asparagine residues. These proteases have been found in *Leishmania* spp., *Trypanosoma cruzi, Schistosoma mansoni*, and *Fasciola hepatica* [20,21,22].

## 3. Biological Functions and Pathogenesis of Cysteine Proteases

### 3.1. Nutrient Metabolism

The most important biochemical property of CP is its proteolytic digestion. The AAs produced by its degradation of host proteins are the main source of nutrients for parasites such as *T. brucei*, *T. vaginalis, Plasmodium falciparum*, *Entamoeba histolytica*, and *Schistosoma*, just to name a few [24,25,26]. In *P. falciparum*, erythrocytic trophozoites hydrolyze the host’s hemoglobin to provide AAs for its own protein synthesis. CP inhibitors block the parasite’s hydrolysis of hemoglobin and hence delay the parasite’s development, indicating that CPs are pivotal for these processes [27,28]. Similarly, *Schistosoma mansoni* uses the host’s hemoglobin as a major source of nutrients and AAs. Degradation of the host’s hemoglobin by *S. mansoni* relies on cathepsins B of its CP family cathepsin B1 (SmCB1) [28,29]. CPs released by *Haemonchus contortus* cultured in vitro for 24 h are similar to cathepsin L and inhibit the agglutination of sheep erythrocytes, hydrolyze hemoglobin, fibrinogen, collagen, and IgG; the hydrolysis of IgG mainly takes places at the hinge region [30]. Moreover, an acidic CP purified from *Paragonimus westermani* with molecular mass 27 kDa of a single-chain polypeptide can degrade human serum albumins, immunoglobulins (Ig), complements, and endogenous protease inhibitors [31]. A 54-kDa CP purified from *Neodiplostomum seoulense* adult worms can degrade extracellular matrix (ECM) proteins such as collagen and fibronectin and participate in the process of the worm’s uptake of nutrients from the host’s intestine [32]. Na and colleagues [33] identified a novel gene encoding a cathepsin F-like CP of *Clonorchis sinensis* (CsCF-6) and characterized its role in nutrient uptake by the parasite.

### 3.2. Invasion of Tissues

Successful invasion of host cells and tissues is an essential step in a parasite infecting a host. Parasites rely on proteases for their migration within a host or host-to-host transfer. Na BK et al. [34] investigated the two distinct isoforms of a 28-kDa enzyme in the newly excysted metacercaria of *P. westermani* (PwNEM28a and PwNEM28b). Both isoforms significantly facilitated the invasion of the newly excysted metacercaria into the murine peritoneum. This invasion process was greatly inhibited by a diffusible CP inhibitor, E-64, in a dose-dependent manner. In addition, the mRNA transcripts of both PwNEM28a and PwNEM28b were abundantly elevated during the active invasion/migration through the host’s tissues. These data collectively show that both PwNEM28a and PwNEM28b are CPs that play an important role in tissue invasion/migration in the definitive host of *P*. *westermani* [34]. The ability of *H. contortus* to degrade connective tissue was studied in vitro using ECM produced by smooth muscle cells. The parasite-secreted CPs degraded the main components of connective tissue; this degradation was beneficial to the parasite infection and destruction of host tissues [35]. Bzik et al. identified a 68-kDa CP from the schizonts and merozoites of *P. falciparum*, which may play a role in invasion of human erythrocytes [36]. Briones et al. also showed that CP is partly involved in the invasion of hepatocytes by *P. berghei* [37]. Moreover, CP secreted by *F. hepatica* had been demonstrated to degrade ECM proteins including laminin, collagen, and basement membrane macromolecules [34,38].

Adherence to host-cell surfaces has been shown to be an early and critical step in the pathogenesis of *T. vaginalis* [39]. Arroyo R [40] showed that pre-treating *T. vaginalis* trophozoites with CP inhibitors, such as N-α-p-toluenesulfonyl-L-lysine chloromethyl ketone (TLCK), Leupeptin, and L-1-tosylamide-2-phenylethyl chloromethyl ketone, etc., greatly reduced their ability to recognize and bind to the epithelial cells of the human genital tract, suggesting that the action of proteases on the parasite surface is a prerequisite for host cell attachment. This was the first time that *T. vaginalis* CPs had been shown to be involved in the attachment of the parasite to human epithelial cells [40]. The first host surface encountered by trichomonads is the mucous layer covering epithelial cells of the human urogenital tract. Mucin, the major proteinaceous constituent of mucous, forms a lattice structure that serves as a formidable physical barrier to microbial invasion. Michael et al. showed that trichomonads can traverse the mucous barrier to the underlying epithelium by binding themselves to mucin followed by its cysteine proteolytic degradation [41]. The mechanism of adhesion to mucins may allow *Trichomonas* trophozoites to gain a temporary foothold before invading the mucus layer and eventually colonizing the underlying epithelial cells [42].

### 3.3. Evasion of the Host Immune Responses

Parasites often live in a host for a long period of time without being recognized and eliminated by the host’s immune system, which is often related to the immune evasion produced by the parasites. CPs contribute to the parasite’s immune evasion of the host through various mechanisms, such as cleaving host’s Ig, blocking antigen presentation, and regulating the secretion of host cytokines and chemokines [43]. For example, an integral part of the immunomodulation of liver fluke *F. hepatica* is the release of CPs. The released CPs cleave host’s Ig, hence reducing or even completely abolishing host antibody-mediated killing and antibody-dependent cellular cytotoxicity (ADCC) against parasites [44]. The cathepsin L-like CPs secreted by *F. hepatica* cleave the host’s Ig, thereby preventing the immune effect of antibody-mediated eosinophil attachment and evading the host’s immune system [45]. A cathepsin S-like CP of 27 kDa secreted by *Spirometra mansion* plerocercoid (Sparganum) can cleave IgG into Fab and Fc fragments, further hydrolyze Fab & Fc fragments, and enhance the degradation by dithiothreitol (DTT), which are all involved in immune evasion used by the parasite [46]. Khalil et al. have demonstrated that larvae of *Taenia crassicep* are able to survive for prolonged periods in the tissues of their intermediate hosts. One possible mechanism of immune evasion is that the host’s Ig, degraded by the enzymatic action of a parasite’s 43-kDa CP, is taken up by the parasite’s cysticerci through adsorptive endocytosis [47]. On the other hand, parasites accelerate the metabolism of their own tegument and tissues through secretion of proteases to hinder their recognition by the host immune system [48]. Cathepsin L has also been shown to inhibit Th1 immune response by inducing IL-4 in animals infected with *F. hepatica*, making them susceptible to concurrent bacterial infections [49]. Current studies have also shown that CPs of *Leishmania amazonensis* and *L. mexicana* amastigotes may be involved in the degradation of the internalized MHC class II molecules [50,51,52], therefore evading the process of antigen presentation and host immune recognition.

Moreover, CPs can eliminate host’s complement-mediated immune effects on parasites to achieve immune evasion [53]. Here, we present a couple of examples. CPs are the most abundant protease in *Entamoeba histolytica*, which can destroy target cells, and cleave and inactivate complement components C5a and C3a, thereby reducing complement-mediated immune responses [54]. In addition, *E. histolytica* trophozoites themselves may evade host’s immune responses by degrading the mucin layer with various glycosidases and CPs [55,56]. Complement regulatory protein (CRP) on the surface of *T. cruzi* trypomastigotes binds to human complement components C3b and C4b, and hence inhibits the formation of C3 convertase; at the same time, the combination of C3b and CRP makes the latter more sensitive to the parasite’s own CPs. Consequently, 3Cb-CRP complexes are cleaved off the surface of the parasite; therefore, these trypomastigotes are capable of avoiding the killing and clearance mediated by complement, leading to immune evasion [57,58].

### 3.4. Cysteine Proteases as Virulence Factors of Parasites

Numerous reports have elaborated that cytotoxicity of parasites depends upon CP activity. CP39 was the first described glycosylated CP of 39 kDa involved in *T. vaginalis* cytotoxicity [59]. Alvarez-Sanchez [60] further demonstrated that *T. vaginalis* 65-kDa CP (CP65) located in the plasma membrane was involved in the cytotoxicity of *T*. *vaginalis* to Hela cell monolayers, serving as a virulence factor. It degraded ECM proteins such as collagen IV and fibronectin, the proteins found in the human vagina. The CP65 had the optimal protease activity under the conditions that often occurred in the urogenital tract of infected patients, i.e., at pH 5.5 at 37 °C. In addition, in a study analyzing the presence of 62-kDa CP and anti-62 kDa protease antibodies in clinical samples from women with *T. vaginalis* infections, symptomatic or not, or without infection, anti-62 kDa antibodies were detected as present in vaginal swabs in 66.6%, 55.5%, and 23.3%, respectively. Further, CP was only found present in vaginal swabs of *T*. *vaginalis*-infected women, suggesting that the 62-kDa proteinase could be a virulence factor as well [61]. *Trichomonas vaginalis* protozoan harbors a double stranded RNA virus of 4.5–5 kb in size named *Trichomonas vaginalis* virus (TVV). TVV has four distinct subspecies TVV1-4. These viruses had been found in numerous *T. vaginalis* clinical isolates originating from a wide spectrum of geographical regions such as the USA, Brazil, Cuba, Czech Republic, Netherlands, Egypt, Kenya, South Africa, China, India, Iran, Philippines, and Taiwan [62]. Importantly, the expression of CPs in the TVV-harboring parasite increases as a hypervirulence phenomenon, affecting disease progression in the human host [63]. Another example of TVVs’ effect on host protein expression is the surface immunogen P270 of *T*. *vaginalis*, although not a CP. This molecule of P270 is located on the cell surface of TVV-positive cells. In contrast, it is mainly in the cytoplasm of TVV-negative cells [64]. Interestingly, TVVs are released from *T. vaginalis* into the small extracellular vesicles and concomitantly modulate the contents of exosomes. The latter specifically included two membrane-associated calpains and 18 soluble CPs of various families, including legumains, calpain-like CPs, and ubiquitin-hydrolase-like CPs [65]. 

The activity of a 24-kDa CP in *C. sinensis* was potentiated 1.9-fold in the presence of 5 mM DTT. Further, its cytotoxicity increased in a dose-dependent manner up to 120 µg/mL. In contrast, the protease’s enzymatic activity was significantly inhibited by N-ethylmaleimide (NEMI) and antipain. Collectively these data suggest that CPs are responsible for cytotoxicity and are closely related to the pathogenicity of *C. sinensis* [66]. CPs secreted by *T. brucei* into the host blood can cause platelet aggregation, which is a serious complication of infection with the parasite [67].

### 3.5. Cysteine Proteases in Immunodiagnosis of Parasitic Diseases

CPs are immunodominant antigens of many parasites with species specificity, so they are good candidates for diagnostic antigens. For example, CP is one of the main components of *C. sinensis* ESP. The positive identification of *C. sinensis* detected by an ELISA using a recombinant CP is persistently consistent with that of the gold standard diagnosis, which is an etiological examination for detecting eggs in a host’s stool. The consistency rate between the two methods was 99.43% [68]. It shows and confirms that recombinant CP has high sensitivity and specificity as immunological diagnosis [69]. Ikeda et al. [70] showed that an ELISA using the natural CP purified from *P. westermani* ESP as antigens not only had higher sensitivity to patients’ sera with paragonimiasis westermani than using the total antigens extracted from adult worms, but also had reduced cross-reactivity with sera with fascioliasis, onchocerciasis, or clonorchiasis to negligible levels. Therefore, this purified CP can be used in immunodiagnosis of paragonimiasis with high specificity and sensitivity. Soon afterwards, Kim et al. [71] identified a 28.5-kDa CP protein of *P. westermani* by generating a recombinant protein. They used degenerate oligonucleotide primers to clone and express the CP gene of *P. westermani* in *Escherichia coli*. Further, Mahmoud [72] used cystatin capture ELISA as a serological diagnosis for human paragonimiasis and schistosomiasis mansoni, and its sensitivity and specificity were as high as 100%, making it an ideal immunological diagnosis for these diseases. 

Immunodiagnosis plays a critical role in control and prevention of fasciolosis, since early detection followed by timely chemotherapeutic intervention and preventing the contamination of water with the parasite eggs is necessary [49]. Cathepsin L proteinase has been used to develop a reliable test for early and specific diagnosis of *Fasciola* infections [73]. In *Toxocara canis*, the recombinant protease (rTc-CPL1), a 30-kDa cathepsin L found in the larval somatic extracts, was applied in immunization of mice and the subsequent antiserum was shown to be highly specific. Further, the sera derived from infected mice and patients contained antibodies against rTc-CPL-1. The study suggested that rTc-CPL-1 may be valuable as a specific diagnostic tool for human toxocariasis [74]. The *Trichinella spiralis* TsATG4B belongs to the C54 peptidase family (Aut2 peptidase family, CA clan) [7]. Studies have shown that TsATG4B is a protease detected by the sera of animals with early *T. spiralis* infection and identified using mass spectrometric analysis. This protease is considered to be a possible immunodiagnostic antigen [75,76].

### 3.6. Cysteine Proteases as a Vaccine Target

The parasite CPs that stimulate strong protective immunity of a host, no matter whether humoral or cell-medicated immunity, are good candidates for vaccines. CP of the nematode *Nippostrongylus brasiliensis* mainly induces humoral responses in host’s production of IgE/IgG1 antibodies [77]. TsATG4B, a CP of *T. spiralis* mentioned previously, is also expected to be a vaccine target in addition to being a possible immunodiagnostic antigen [75,76]. The target calcineurin for the protective immunity of *S. mansoni* has been used as a vaccine in a murine model, and CD4+ T lymphocytes using the calcineurin epitope as the target antigen can be passively transferred; this passive immunity provides the recipients with persistent protection against *S. mansoni* infection [78]. Two CPs (Fas1 and Fas2) were purified from ESP of adult *F. hepatica*. Western blot analysis showed that they are immunodominant antigens that can be recognized with high sensitivity and specificity by the sera of patients with *F. hepatica* infection [72]. Further, cattle immunized with these CPs showed protection against *F*. *hepatica* infection, as demonstrated by decreased infection rate, reduced number of eggs in the excrement, and diminished viability of the eggs in comparison with unimmunized controls [79,80]. The lambs immunized with the membrane protein extracts of adult *H. contortus*, which is rich in CPs, followed by a challenge infection showed that female-worm ovulation and total worm load decreased by 77% and 47%, respectively, compared with those of the unimmunized controls [81,82]. BALB/c mice immunized with 30-kDa CPs of *L. amazonensis* showed some protection against amastigote infection due to Th1 cell-related immune responses [83]. Protection due to humoral responses to CPs of various parasites has been observed and reported, such as in *E. histolytica* and *S. mansoni* [84], and in *T. cruzi* [85] and *L. mexicana* [86]. All these lay a solid foundation in both theory and practice for CPs to be considered as candidate antigens for vaccine development.

### 3.7. Cysteine Proteases as New Targets for Chemotherapy of Parasitic Diseases

As mentioned earlier, CPs play extremely important roles in parasite’s nutrient uptake, invasion, immune evasion, and pathogenicity to the host. Logically, they can be an excellent target for chemotherapy of parasitic diseases [87,88,89,90]. As a matter of fact, the successful treatment of parasitic diseases by inhibiting CP activities in animal models has been proved effective in principle [91,92,93,94]. Here, we present a few examples. The first is *Schistosoma* spp. Wasilewski et al. [26] showed that two different types of irreversible specific CP inhibitors effectively blocked the degradation of host hemoglobin by *Schistosoma* parasites in vitro, resulting in their death within 7 days. Further, infected mice with early infection upon one-week treatment showed a significant reduction in worm burden. Alternatively, Jilka et al. [95] confirmed that *S. mansoni* cathepsin B1 (SmCB1) is a validated drug target, and inhibitors of SmCB1 represent a promising treatment of schistosomiasis as well.

The second example is *P. falciparum*. Falcipain, a CP of *P. falciparum*, is required for the degradation of the host’s hemoglobin [96]. Peptidyl inhibitors of falcipain blocked hemoglobin degradation by parasites and inhibited parasite development. One of these inhibitory compounds, when administered parenterally, cured *P. vinckei*-infected mice [97]. Moreover, it has been shown in a murine model that peptidyl inhibitors of falcipain, such as fluorescein ketone and vinyl sulfone, successfully cured malaria in mice [98]. 

### 3.8. Cysteine Proteases as Targets for Genetic Manipulations

Drug resistance and side effects make the treatment of parasitic diseases more and more challenging. Millions of people worldwide are still at risk of parasitic protozoan infections, resulting in more than one million deaths each year [99]. This situation mandates a shift in our attention to new targets primarily for therapeutic, diagnostic, and research applications. CP is one such new target. CRISPR (clustered regularly interspaced short palindromic repeats), a new genetic manipulation tool, provides excellent opportunities for genetic manipulation of genes encoding CPs. A CRISPR-based diagnostic method has been developed for the detection of *Plasmodium* spp. with ultra-sensitivity that is suitable for individuals with malaria parasite infections, no matter whether they are symptomatic or not [100]. 

Not all genetic manipulations use CRISPR. They may be achieved by homologous recombination. CP falcipain-2 of *P*. *falciparum* has been proven to be crucial for the parasite in its hydrolysis of the host’s hemoglobin, as demonstrated by knocking out its gene [101]. Another CP falcipain-3 of *P*. *falciparum* is proven to be required for the erythrocytic parasites as well, since an attempt to knock it out failed [102]. Calpain belongs to the C2 family CPs of *P*. *falciparum*. The notion that it is necessary for the parasite growth is supported by the failure of gene disruption by double cross-over and gene truncation by single cross-over recombination. It is confirmed that this CP is necessary for cell cycle progression, especially in pre-S-phase development in *Plasmodium* spp. parasites [103]. Last, but not least, disruption of cathepsin protease L, a CP of *T*. *gondii* protozoa, impairs the digestive function of the organelle called vacuolar compartment, and greatly diminishes chronic infections of the parasite. Chronic infection is the most important feature of *T. gondii* infection of almost all warm-blooded animals [104].

## 4. Conclusion Remarks

**Figure 3:**
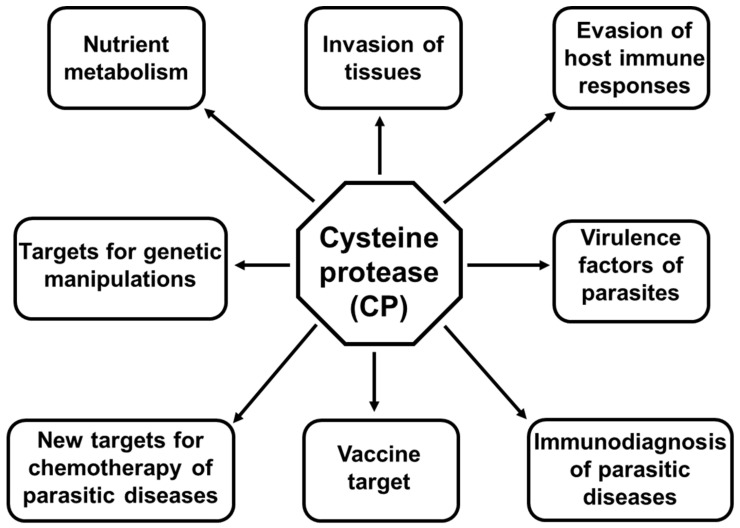
Biological functions and pathogenesis of cysteine proteases in unicellular and multicellular endoparasites and parasitic infections.

Cysteine proteases exist ubiquitously in all living organisms. They are classified into 113 families in ten well-defined clans. They play pivotal roles in the biology of all parasitic organisms, ranging from nutrient acquisition to evasion of host immune response, and serve as virulence factors for pathogenesis (Figure 3). The more we know about them, the better diagnosis, treatment, control, and prevention of parasitic diseases in the near future will be. Therefore, more attention must be paid to these pivotal molecules of various endoparasites, especially those that are released by exosome and ESP, since they are capable of interacting with targets that are located not only nearby but also at distance.

## Data Availability

All data generated or analyzed during this study are included in this published article.

## Abbreviations

AA: amino acid; CP: cysteine proteases; CRIPR: clustered regularly interspaced short palindromic repeats; CRP: complement regulatory protein; DTT: dithiothreitol; ECM: extracellular matrix; ESP: excretory-secretory proteins; GPI: glycosylphosphatidylinositol; Ig: immunoglobulin; PV: parasitophorous vesicles; TVV: *Trichomonas vaginalis* virus.
